# Immunogenetics of Small Ruminant Lentiviral Infections

**DOI:** 10.3390/v6083311

**Published:** 2014-08-22

**Authors:** Nancy Stonos, Sarah K. Wootton, Niel Karrow

**Affiliations:** 1 Centre for the Genetic Improvement of Livestock, Department of Animal and Poultry Science, University of Guelph, Guelph, ON N1G 2W1, Canada; E-Mail: nstonos@uoguelph.ca; 2 Department of Pathobiology, Ontario Veterinary College, University of Guelph, Guelph, ON N1G 2W1, Canada; E-Mail: kwootton@uoguelph.ca

**Keywords:** SRLV, immune response, genetic selection

## Abstract

The small ruminant lentiviruses (SRLV) include the caprine arthritis encephalitis virus (CAEV) and the Maedi-Visna virus (MVV). Both of these viruses limit production and can be a major source of economic loss to producers. Little is known about how the immune system recognizes and responds to SRLVs, but due to similarities with the human immunodeficiency virus (HIV), HIV research can shed light on the possible immune mechanisms that control or lead to disease progression. This review will focus on the host immune response to HIV-1 and SRLV, and will discuss the possibility of breeding for enhanced SRLV disease resistance.

## 1. Introduction

The caprine arthritis encephalitis virus (CAEV) and the Maedi-Visna virus (MVV) are enveloped RNA viruses in the lentivirus genus of the Retroviridae family [1,2]. While small ruminant lentiviruses (SRLVs) were once considered to be species-specific, recent studies suggest that they can be transmitted between sheep and goats [3], and can recombine to form new CAEV-MVV strains [4]. These viruses primarily infect monocytes, macrophages, and dendritic cells [5], and like the human immunodeficiency virus (HIV), infection is lifelong and can persist for months or years in a latent or sub-clinical state [6]. When disease symptoms do emerge, goats predominantly show signs of arthritis or mastitis, while in sheep the disease tends to manifest as pneumonia or mastitis [7]. Encephalitis can also be a symptom in either lambs or kids but is less common [8].

SRLVs are predominantly vertically transmitted to offspring through the shedding of virus particles, and infected macrophages and epithelial cells in the colostrum and milk [9]. Horizontal transmission however, can occur through prolonged direct contact with bodily secretions, and sexual transmission of the virus may also be possible [10]. There is currently no effective treatment for SRLV infections and due to a high mutation rate, effective vaccine development has been and will continue to be challenging [11]. Therefore, the most effective means of controlling the virus is through herd management that prevents viral transmission.

The dynamics of the host immune response to SRLV infections remain unclear, but due to the similarities between SRLV and HIV, a great deal of our knowledge of the immune responses to HIV can be used to enhance our understanding of the host response to SRLV. This review will discuss the immune response to lentiviral infections, and the possibility of breeding for enhanced SRLV resistance will be addressed.

## 2. Lentiviral Characteristics

To fully understand the complex interaction between the host and virus, it is first necessary to understand the structural and genomic organization of the lentiviruses. Although an in-depth discussion of the structural and functional characteristics of lentiviruses is beyond the scope of this paper, a brief overview of SRLV and HIV-1 organization is given.

### Genomic Organization

**Figure 1:**
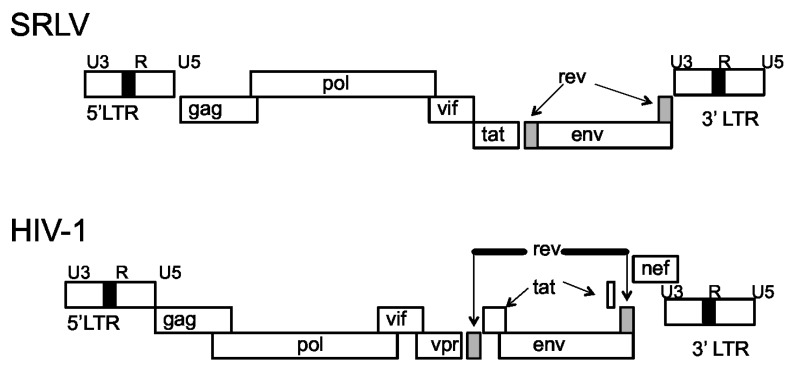
Genomic organization of small ruminant lentiviruses (SRLV) and HIV-1 [23].

The SRLV genome is comprised of three structural genes, *gag*, *pol* and *env*, and three accessory genes, *vif*, *tat*, and *rev* (Figure 1) [12,13]. The *gag* gene encodes the capsid proteins, *pol* encodes the viral enzymes protease, reverse transcriptase, and integrase, and *env* encodes the envelope glycoproteins, gp135 (SU) and gp38 (TM) [14]. While the *tat* gene is dispensable for efficient viral replication [15], *Vif* is absolutely required for efficient *in vivo* virus replication and pathogenicity [16,17]. The flanking ends of the proviral DNA are regions of long terminal repeats (LTRs) that are divided into the U3, R, and U5 regions [18,19,20]. These regions provide the signals required for viral transcription and integration into the host genome [19]. Small ruminant lentiviruses differ from the primate lentiviruses in that their Tat proteins do not *trans*-activate the viral LTR promoters [21]. Rather, the SRLV Tat protein is functionally similar to the HIV type 1 (HIV-1) Vpr protein [22].

The HIV-1 genome is similar to the SRLV genome, but is more complex and contains additional genes [23]. In contrast to SRLV, HIV-1 contains nine genes encoding 15 proteins; these genes, include the structural *gag, pol*, and *env* genes that encode the capsid proteins, the viral enzymes, and the envelope glycoproteins, gp120 (SU) and gp41 (TM), respectively [24]. The HIV-1 non-structural genes consist of *vif*, *tat*, *rev*, *nef*, *vpu*, and *vpr*, and are associated with HIV-1 pathogenesis and immune evasion [25]. Like the SRLV genome, the flanking ends of the provirus contain LTRs consisting of U3, R, and U5 regions [26]. Additionally, a variety of promoter and enhancer elements have been identified in the LTR region of HIV-1. Some of these response elements include AP-1, NF-κB, and Sp1 [27], important host immune related transcriptional factors which all lead to transcriptional activation and virus replication.

## 3. Host Immunity to Lentiviral Infections

There are substantial gaps in our knowledge of host innate and acquired immune responses to SRLV. Due to similarities between HIV-1 and SRLV, HIV-1 research can improve our understanding of SRLV immune responses. This section will provide an overview of the immune response to HIV-1, and discuss the current knowledge of the immune response to SRLV infection.

### 3.1. The Innate Immune Response to HIV-1

#### 3.1.1. Toll-like Receptors and Antiviral Peptides

Toll like receptors (TLR) are host pattern recognition receptors (PRRs) that play a key role in innate recognition of a variety of conserved pathogen-associated molecular patterns (PAMPs). Ligation of PAMPS with PRRs induces intracellular signaling pathways that involve a series of phosphorylation events mediated through the common adaptor molecule, MyD88, reviewed by [28]. Downstream effects of these signaling pathways result in the activation and translocation of NF-κB or AP1 transcription factors to the nucleus, and subsequent pro-inflammatory cytokine production that facilitates the recruitment of innate effector cells such as neutrophils and macrophages to clear the pathogen [29].

During HIV-1 infection, the roles of TLR 7 and 8 have been investigated. Both TLR7 and 8 recognize single-stranded viral RNA (ssRNA), and this results in the induction of intracellular signaling cascades that involve NF-κB transcriptional activation, which ultimately results in the production of a variety of pro-inflammatory cytokines such as the type 1 interferons (IFN-α and -β), IL-6, and TNF-α [30,31]. Although this serves as an anti-viral defense mechanism through the induction of antiviral peptides such as APOBEC3G (A3G), tripartite motif 5-alpha (TRIM5α), and tetherin, persistent immune activation also induces viral replication through the activation of the NF-κB response element located within the HIV-1 LTR [27]. HIV-1 replication can also be induced through the activation of other TLR signaling pathways. For example, Ranjbar *et al.*, [32], observed that HIV-1 replication can also be induced through activation of TLR2 during Mycobacterium tuberculosis infection. Since the recruitment of MyD88 and activation of either NF-κB or AP1 is conserved across TLRs 1, 2, 4, 5, 6, 7, 8, and 9 [28], recognition of a variety of bacterial and viral PAMPS may all contribute to HIV-1 transcriptional activation and disease progression.

Despite the induction of HIV-1 replication mediated through TLR signaling, there are host-adapted antiviral proteins that serve to control HIV-1 replication. Expression of these antiviral peptides is upregulated by type 1 interferons that are induced in response to TLR activation [33]. TRIM5α, for example, is a lentivirus restriction protein that mediates cellular restriction against retroviruses in a species-specific manner [34]. Although human TRIM5α poorly restricts HIV-1, polymorphisms within the human TRIM5α gene have allowed for the development of human TRIM5α with high HIV-1 restriction activity [34]. The exact mechanism by which TRIM5α works is not well understood, however, it does concentrate around the viral core and recognizes and binds to the viral capsid to facilitate rapid uncoating [35]. This disrupts the reverse transcription process since uncoating is a carefully regulated process, and early or delayed uncoating may negatively affect virus infectivity [36]. Once the capsid has been targeted by TRIM5α, the viral core is disassembled which is likely mediated by proteosomal association with TRIM5α-virus complexes [36].

A3G is another antiviral protein expressed by lymphocytes, macrophages and dendritic cells following cellular stimulation by IFN-α and IL-2 [37]. A3G restricts HIV-1 infection by two separate mechanisms. The first involves the packaging of A3G during viral assembly directly into viral capsids in the absence of vif, and this occurs through the interaction with the viral capsid proteins [38]. This provides A3G with direct access to the HIV-1 genome where it induces mutations during the reverse transcription process by editing cytosine residues to uracil residues in the proviral minus strand [37,38,39]. The mutated viral DNA is then degraded due to reduced stability or the inability to be incorporated into the host genome [40]. However, these mutations are not always sufficient to prevent proviral integration, if the viral DNA still integrates successfully, the mutations often alter viral open reading frames or introduce premature stop codons resulting in misfolded or truncated viral proteins that are unable to produce infectious particles [37,40]. These misfolded viral proteins provide host cells with a pool of viral peptides that can be presented onto major histocompatibility complex (MHC) I molecules for antigen presentation. Expression of A3G may also enhance MHC I presentation and promotes the activation of cytotoxic T lymphocytes (CTLs) [37]. The other mechanism by which A3G limits HIV-1 infectivity is by directly interfering with the reverse transcription process, which may occur through the direct interaction between A3G and the viral reverse transcriptase enzyme [41]. Although A3G is highly effective at controlling virus replication, HIV-1 has adapted mechanisms to counteract the effects of A3G [37,40]. The HIV-1 accessory gene, *vif*, is essential for virus replication, but also has a role in promoting A3G degradation [25,37,40]. *Vif* binds A3G and mediates its polyubiquitination, tagging it for proteosomal degradation [39], but may also inhibit A3G mRNA translation by directly binding A3G mRNA to alter its stability [39].

Tetherin is also induced by type 1 IFNs and restricts HIV-1 by preventing virion release from the cell surface [42]. Since tetherin is an integral membrane protein with cytoplasmic, transmembrane, and extracellular domains, it can be incorporated into the membrane of virion particles as they bud from the cell surface [43]. Consequently, this serves to anchor virion particles to both the cell surface and to other virion particles as they bud from the host cell [42]. Once anchored to the cell membrane, virion particles can be endocytosed and degraded in lysosomal compartments [44]. Interestingly, HIV-1 has adapted a mechanism to prevent viral tethering. The HIV-1 *vpu* gene for example, encodes an integral membrane protein that interacts with tetherin transmembrane domains [45]. The vpu protein prevents the incorporation of tetherin into the envelope of virion particles and down regulates tetherin expression at the cell surface by trafficking tetherin to the trans-golgi network and away from the sites of virion assembly prior to lysosomal degradation [45].

Other antiviral proteins include SAMHD1 and the zinc finger antiviral protein (ZAP). SAMHD1 is a host protein found in resting macrophages, dendritic cells, and CD4^+^ T cells that cleaves deoxynucleoside triphosphages (dNTP) into deoxynucleosides and inorganic triphosphates, which depletes the dNTP pool required for HIV-1 reverse transcription [46]. This prevents the synthesis of full-length double stranded viral DNA and therefore prevents integration of proviral DNA [46]. The *vpx* gene of HIV-2 and the simian immunodeficiency virus (SIV) has the ability to disrupt SAMHD1 by interacting with the C-terminus and promoting proteosomal degradation [46]. HIV-1, however, does not contain the *vpx* gene so HIV-1 replication is actively suppressed in resting CD4^+^ T cells [47]. ZAP has been identified for its role in restricting the murine leukemia virus (MLV) along with HIV-1, however, some viruses can replicate normally in ZAP-expressing cells [48]. ZAP restricts HIV-1 by depleting multiply spliced mRNA by recruiting poly(A)-specific ribonucleases that shorten the poly(A) tail and directs mRNA to exosomes for degradation [48].

#### 3.1.2. Natural Killer (NK) Cells

Further research investigating the innate control of HIV-1 has focused on identifying the roles of natural killer (NK) cells in controlling viral replication. The exact role of NK cells during HIV-1 infection is not well understood, however, it has been suggested that NK cells serve as a means of controlling viral replication prior to the induction of HIV-1-specific CD8^+^ T cell responses [49]. NK cells target HIV-1 infection by directly killing infected cells through the killer immunoglobulin-like receptor (KIR)-mediated recognition of target cells, degranulation resulting in granzyme and porforin release, the Fas-Fas ligand pathway, antibody-dependent cell-mediated cytotoxicity (ADCC), and modulating adaptive immune responses with IFN-γ production [50]. During acute HIV-1 infection, NK cells are known to rapidly proliferate [49], however, these cells may not be fully functional [48]. For example, Naranbhai *et al.*, [51] observed reduced cytotoxic NK cell responses during acute HIV-1 infection. Similarly, antibody dependent cell mediated cytotoxicity (ADCC), the process by which antibodies bind to a specific pathogen and subsequently crosslink with Fc receptors on NK cells, macrophages, neutrophils, and mast cells, is also impaired during acute HIV-1 infection [50]. However, this may be attributed to the time required to generate HIV-1 specific antibody levels [50]. Once sufficient HIV-1 specific antibody levels were generated, strong NK cell responses were observed in chronic HIV-1 infected patients [52]. Although reduced cytotoxic responses and ADCC may be impaired during acute infection, the proportion of activating and inhibitory receptors on NK cells appears to be elevated [50]. As the disease progresses, a lower ratio of inhibitory to activating receptors has also been observed [50]. NK target cell lysis involves a balance between activating and inhibitory signals mediated through the ligation of MHC class I molecules. An increase in activating receptors during chronic HIV-1 infection implicates NK cells as important effector cells for controlling disease progression, however, further investigation into the diverse role of NK cells during HIV-1 infection is warranted.

#### 3.1.3. γδ T Cells

γδT cells are a unique subset of innate immune effector cells that possess the γδT cell receptor. Unlike CD4^+^ and CD8^+^ T cell subsets, γδT cells do not require antigen presentation to become activated. The γδT cell subset can be further divided into Vδ1 and Vδ2 cells, which are localized to mucosal surfaces and peripheral blood, respectively [53]. Little is known about the role of γδT cells in HIV-1 infection; however, they do appear to play an important role in the control of HIV-1 viral replication. For example, Fenolilo *et al.*, [53] observed an expansion of Vδ1 cells during HIV-1 infection, and these cells contained elevated gene expression levels for IFN-γ and IL-17. Similarly, in African patients, Vδ1 cells appeared to be expanded during HIV-1 and HIV-2 infection, and Vδ1 T cell counts appeared to be positively correlated with CD4^+^ T cell counts [54]. In addition to their cytotoxic activity and pro-inflammatory cytokine production, γδT cells have also been identified for their role in ADCC. *In vitro* studies have suggested that Vδ2 cells from HIV-1 patients are expanded and have potent ADCC activity [55]. Given that Vδ2 cells are present in the circulation, they have direct access to both circulating antibodies and circulating HIV-1 virion particles and may prove effective at controlling HIV-1 dissemination throughout the body.

### 3.2. Acquired Immunity to HIV-1

The acquired immune response involves cell-mediated (CMIR) and antibody-mediated immune responses (AbMIR). A balance between both responses is essential to maintaining overall health. The CMIR is primarily designed to combat intracellular pathogens such as viruses, and intracellular bacteria and parasites, whereas, the AbMIR has developed to combat extracellular pathogens such as extracellular bacteria and parasites. The CMIR involves antigen uptake and presentation by professional antigen presenting cells (APC) such as dendritic cells (DC) and macrophages that reside within epithelial cell layers. Following antigen uptake, the DCs migrate to a draining lymph node where they produce IL-12 to promote Th1 cell differentiation, and present antigens via MHC class II molecules to CD4^+^ T helper cells. This allows CD4^+^ T cells to become activated and produce the Th1 subset of cytokines, IFN-γ and IL-2. Production of IL-2 by CD4^+^ T cells is particularly important as it is necessary for the development and expansion of antigen-specific CD8^+^ T cells that recognize foreign antigen in the context of MHC class I molecules as a means of killing infected cells. The Th1 cytokine profiles also induce immunoglobulin (Ig) G2 production by B cells, which promotes complement activation and opsonization [56].

The AbMIR also involves antigen processing and presentation by DCs to CD4^+^ T cells, however, different cytokine profiles allow for the differentiation of Th2 cell subsets. These key cytokines include IL-3, 4, 5, 9, 10, and 13, which serve to suppress Th1 immune responses. In order to produce high levels of antigen specific antibodies, B cells must first be primed during initial antigen exposure. During this primary response, B cells differentiate and expand into memory or plasma cells. The plasma cells initially secrete IgM and small amounts of IgG. Upon subsequent antigen exposure, memory B cells become activated to rapidly produce high levels of IgG1 and other antibody isotypes.

Immune responses to HIV-1 infection tend to vary greatly from individual to individual; therefore, HIV-1 patients are generally classified based on their ability to control infection [57]. Although there is no standardized viral set point or CD4^+^T cell level to define a HIV-1 controller or progressor, in general, a controller is able to maintain low viral loads and CD4^+^ T cell levels in the absence of highly active antiretroviral therapy (HAART); whereas HIV-1 progressors have high viral loads and low CD4^+^ T cell counts [58]. However, many of the factors that differentiate a controller from a progressor are unclear, and despite over 30 years of HIV-1 research, there is still a great deal that is largely unknown.

#### 3.2.1. Cell-Mediated Immune Response to HIV-1

The CMIR to HIV-1 is unique in that HIV-1 preferentially infects CD4^+^ CCR5^+^ T helper cells. During acute HIV-1 infection, the virus rapidly replicates and CD4^+^ T cell population numbers decline [59]. This decline in CD4^+^ T cells is attributed to both direct killing by the virus, and targeted CD8^+^ cytotoxic T lymphocyte (CTL) responses [59]. Maintenance of CD4^+^ T cells levels is essential to maintain HIV-1 specific CTL responses, which is associated with control of the infection [60]. During acute HIV-1 infection, HIV-1 specific CTLs emerge prior to neutralizing antibodies [61]. However, robust CTL responses to HIV-1 may not necessarily control the infection, as the viral nef protein is known to down-regulate MHC class I molecules to evade CTL responses [62]. It has also been suggested that CTL-mediated control of HIV-1 is specific to certain HIV-1 peptides. CTLs have been observed to mount the strongest cytotoxic responses to the gag and nef peptides [63,64]. However, this also induces selective pressure on HIV-1 to mutate these regions creating viral escape mutants [65]. It has been suggested that these HIV-1 escape mutants may be associated with the inability to control infection; however, this association is not always clear [66]. Some studies have suggested that these mutations come at a fitness cost to the virus [61,67,68], and escape tends to occur rapidly during acute infection and declines as the infection reaches the chronic state [61,69]. In some instances, the rate of mutation occurs so rapidly that after the initial infection, the transmitted virus or founder virus is completely lost [61]. Therefore, CTL responses that occur during acute infection must continuously adapt to changing HIV-1 peptides [61,70]. As the infection becomes chronic, immunological exhaustion becomes apparent due continuous immune activation in response to viral replication [71,72]. Consequently, CD8^+^ CTLs often exhibit an exhausted phenotype with increased CTLA-4 and PD-1 receptor expression and reduced cellular function [71,73]. HAART can help prevent this by limiting viral replication, however, if left untreated, the disease will continue to progress [73].

#### 3.2.2. Antibody-Mediated Immune Response to HIV-1

Antibody responses to HIV-1 infection do not emerge until approximately 13 days post infection [74]. These antibodies include gp41-specific IgM and IgG, which are non-neutralizing [74]. As the infection progresses, IgG1 specific gp120 antibodies are produced [75]. These antibodies are specific to a variety of epitopes on gp120 including the CD4 binding site, glycan-containing regions, and the V3 loop [75]. The early production of non-neutralizing antibodies has little effect on viral infectivity and control of viral load; however, they do play a role in ADCC and viral opsonization [76]. During acute HIV-1 infection, IgG1-virus immune complexes are predominately comprised of gp41 antibodies and are present at relatively low levels compared to the viral load [76]. In contrast, during chronic HIV-1 infection, these virus-immune complexes were more abundant and are comprised of gp120-specific IgG1 [76,77]. This suggests that although HIV-1 specific antibodies emerge early after infection, they are not effective at controlling the viral load [76]. Neutralizing antibodies begin to emerge as early as 16 weeks post infection [75]. However, these early neutralizing antibodies may not be fully functional, as broad neutralizing ability does not emerge in HIV-1 patients until 2–3 years post infection [75]. Some HIV-1 infected patents, in contrast, never develop neutralizing antibodies, and it is unclear what factors contribute to these differences [78]. Additionally, the emergence of neutralizing antibodies may not necessarily control the infection. For example, Mikell *et al.* [78] observed a positive correlation between neutralizing ability and viral load, and Euler *et al.* [79] observed lower percentages of CD4^+^ T cells in patients with strong neutralizing activity; suggesting that HIV-1 may mutate env epitopes to escape virus neutralization.

#### 3.2.3. Other T Cell Subsets

Recent studies have implicated roles of Th17 and Treg cells in the pathogenesis of lentiviral infections. Research investigating the role of Th17 cells in HIV-1, for example, found that long-term non-progressors had higher Th17 cell numbers compared to disease progressors, and higher Th17 cell numbers were associated with a lower viral load [80]. Additionally, it has been established that Th17 cells and Treg cells interact during HIV-1 infection to regulate immune responses [81]. Long-term non-progressors tended to have Th17/Treg ratios similar to uninfected controls, whereas, individuals that did not easily control the rate of viral replication had depleted Th17 numbers and increased Treg numbers suggesting a switch towards an anti-inflammatory immune response [81]. However, it is unclear if maintenance of Treg cell populations during HIV-1 infection is associated with rapid or delayed disease progression. It has been suggested that Treg-mediated IL-10 production is also associated with HIV-1 disease progression as it suppresses specific CD4^+^ T cell responses [82]. However, other studies have suggested that high levels of Treg cells can help prevent disease progression by limiting CD4^+^ T cell and CTL responses, thus limiting continuous immune activation and preventing immunological exhaustion [83,84]. Therefore, further research is required to understand the role of Tregs in HIV-1 disease progression.

### 3.3. Innate Immune Response to SRLV

#### 3.3.1. Toll-Like Receptors and Antiviral Peptides

The role of viral-induced TLR signaling has not been widely studied in sheep and goats, however, during SRLV infection, TLR 7 and 8 become activated inducing IFN-α, IL-6, TNF-α production and subsequent antiviral protein expression [85]. It is unclear if TLR signaling pathways induce SRLV replication, or if the SRLV genome has a NF-κB transcriptional binding site in the promoter. However, given the importance of macrophages as innate immune effector cells, macrophage maturation and activation can induce SRLV replication.

There is considerably less research investigating the roles of the intrinsic restriction factors TRIM5α, A3G, and tetherin in SRLV infection, however, TRIM5α has recently been identified in sheep and goats, and has been found to be effective at restricting SRLV [86]. An A3G- like protein has also been identified in sheep, and has shown cytodine deaminase activity [87]. Like HIV-1, SRLV contains the accessory *vif* gene to combat the restrictive activity of A3G, and SRLV *vif* appears to restrict A3G across species [87]. Tetherin has been investigated in sheep due to its role in restricting endogenous retroviruses [88]. Since the SRLV genomes lack the accessory gene *vpu*, tetherin likely has high SRLV restriction activity. However, further investigation into the roles of TLR activation and intrinsic restriction factors in limiting SRLV infection is necessary.

#### 3.3.2. NK Cells

The role of NK cells in SRLV infection has not been investigated; however, given the importance of NK cells for HIV-1 infection, it is likely that they play an important role in the control of SRLV. It is unclear how NK cells target SRLV-infected macrophages; however, we may speculate that they recognize and bind infected cells and virion particles through a number of mechanisms including KIR-mediated recognition, degranulation, complement activation, ADCC and the production of IFN-γ which serves to either kill infected cells, or modulate virus-specific immune responses. ADCC has been investigated as a possible control mechanism in MVV infected sheep [89]. Sheep vaccinated with a recombinant env protein had higher IgG2 antibody titers an IgG1 and polyclonal serum had ADCC activity compared to MVV-infected non-vaccinated animals suggesting that MVV specific IgG2 may have strong ADCC activity early in infection [89].

#### 3.3.3. γδ T Cells

In ruminants, γδT cells comprise approximately 70% of all lymphocytes in young animals, and are an important part of the innate immune system [90]. CAEV-infected goats have a significantly higher proportion of γδT cells compared to healthy goats, which suggests that these cells may be important for controlling SRLV infection [90,91,92]. Given that γδT cells tend to localize to mucosal surfaces, it is possible that this cell type plays a crucial role in limiting SRLV entry and mediating early immune responses against these viruses.

### 3.4. Acquired Immunity to SRLV

During SRLV infection, both branches of the acquired immune system are activated though it remains unclear how each relates to either host protection or disease progression [93]. Like HIV-1, the degree of the immune response influences the viral load, which is correlated to the severity and presence of clinical disease symptoms [94,95]. Animals that respond with a CMIR are often referred to as long-term non-progressors because they exhibit a persistent viral infection but lack clinical symptoms and have a low viral load. These animals produce high levels of IgG2 antibodies specific to gp135, and a dominant subset of gp135 responsive Th cells displaying high levels of IFN-γ gene expression [94,95]. In contrast, arthritic animals tend to mount a type 2 or AbMIR, characterized by high polyclonal SRLV reactive IgG1 antibody titers and a dominant subset of Th2 cells with low proliferation and enhanced IL-4 gene expression [84,95].

#### 3.4.1. Cell-Mediated Immune Response to SRLV

The CMIR is likely the most efficient response for controlling viral load. Since SRLV, unlike HIV-1, does not infect CD4^+^ T cells, the maintenance of CD4^+^ T cell populations during SRLV infection will allow for the development and maintenance of SRLV-specific CTLs. However, SRLV may interfere with CD4^+^ T cell proliferation as CAEV-infected arthritic goats had reduced CD4^+^ T cell proliferation compared to CAEV-infected asymptomatic goats [96]. Reduced lymphocyte proliferation was also observed in clinically affected sheep compared to MVV-infected asymptomatic animals [97]. Since the SRLV genome does not contain the viral *nef* gene, down-regulation of MHC I by SRLV likely does not occur. However, SRLV may down-regulate MHC class II molecule expression on SRLV-infected macrophages [91], and down-regulation of CD80 co-stimulatory molecules has been observed in sheep with clinical disease symptoms [97]. Overall, this suggests that SRLV infection appears to interfere with antigen processing and presentation, and thus limits the ability of antigen presenting cells to activate CD4^+^ T cells and induce CTL responses.

Although the CMIR is the preferential response for maintaining a low viral load, the presence of type 1 cytokines is not sufficient to control viral replication [94]. In fact, some of the Th1 cytokines, including IFN-γ, TNF-α and GM-CSF, activate the SRLV promoter and induce viral replication [98]. These cytokines activate the viral promoter in the U3 region 70 base pair repeat and this is mediated through the STAT1 pathway [99]. This also requires at least one gamma-activating site (GAS) within the viral promoter [100], indicating that monocyte differentiation and macrophage activation can induce viral transcription. Although not all SRLV strains contain a GAS, the presence of a GAS in the SRLV LTR is not necessary for viral replication [101]. However, high levels of viral replication in response to Th1 cytokines may lead to a cycle of continuous immune activation and to eventual immunological exhaustion and disease progression.

#### 3.4.2. Antibody-Mediated Immune Response to SRLV

The antibody response to SRLV generally targets epitopes on the gp135, gp38, and capsid proteins [102]. Antibody responses can emerge as early as 2–4 weeks post infection, and tend to fluctuate during the first 6 months of infection [103]. Additionally, like HIV-1 these early antibodies are specific to linear epitopes and are thus non-neutralizing [94,96]. However, as discussed previously, these early antibodies may play an important role in ADCC [89]. Neutralizing antibodies can take as long as 2 years to emerge and can control virus infection [104]. However, like HIV-1, SRLV virus epitopes may mutate in response to selection pressure imposed by host immunoglobulins [104,105]. These mutations tend to occur in the fourth variable domain of gp135 and the mutation of a conserved cytosine was shown to change the neutralization epitope [106], these mutations likely contribute to disease progression. Antibody responses, in general, may contribute to SRLV disease progression, since asymptomatic animals also tend to mount a CMIR with a low titer of gp135-specific IgG2, while arthritic animals exhibit very high levels of IgG1 and a higher IgG1/IgG2 ratio than asymptomatic animals [107]. Although further research is necessary to better understand the roles of neutralizing antibodies in the control of SRLV infection, it is evident that an AbMIR is not sufficient to control infection [108], and readily contributes to disease progression.

#### 3.4.3. Immune Dysregulation

The dynamics of immune evasion strategies of SRLV are unclear, however, SRLV infection can alter macrophage function, and immune dysregulation is apparent. This is particularly evident in animals that exhibit clinical signs of arthritis or mastitis, as these inflammatory conditions are characterized by dense mononuclear cell infiltration accompanied by necrosis and edema [108]. Arthritic animals also exhibit thickening and fibrosis of the articular capsule, and erosion and ulcer formation of the articular cartilage occurs in severe cases [109]. Consequently, lesions form in the joint synovial membranes as well as the mammary gland [5]. Histological analysis of these lesions revealed large numbers of macrophages, CD8^+^ T cells and B cells, and the proportion of B cells present in the lesions increases as the infection persists [110]. To investigate the immune dysregulation that occurs during SRLV infections, Lechner *et al.*, [111] examined the cytokine profiles produced by CAEV-infected macrophages in culture. This study revealed that infected macrophages produced elevated IL-8 and monocyte chemotactic protein-1 (MCP1), but had reduced levels of TGF-β mRNA [101]. In addition to this, reduced levels of TNF-α, IL-1β, IL-6 and IL-12 mRNA, and increased levels of GM-CSF mRNA were observed in lipopolysaccharide (LPS)-stimulated CAEV-infected macrophages [111]. Higher levels of pro-inflammatory cytokine gene expression, such as IL-1β, IL-6, IL-12, and TNF-α likely contribute to increased immune activation and trafficking of effector cells to the infection site causing inflammation and lesion formation. Elevated levels of GM-CSF has also been observed in alveolar macrophages of MVV infected sheep [112], and SRLV infection can induce a phenotypic shift in macrophages from pro-inflammatory M1 to an anti-inflammatory M2 macrophages that favoured viral replication [113]. Interestingly, several reports have suggested that SRLV-infected goats are not immunocompromised [5,6,111,114], as is the case for HIV-1 patients. However, the altered cytokine profiles observed in CAEV- and MVV-infected macrophages, along with the reduced DTH response to mycobacterial antigens in MVV-infected sheep, suggest that altered cellular functions may directly affect immunity [115].

## 4. Genetics of Lentiviral Resistance

There is little research investigating the genetic parameters that affect resistance or susceptibility to SRLV, however, research with HIV-1 patients has led to the discovery of a variety of genetic polymorphisms that appear to be associated with disease resistance, or slower disease progression. Most of the genes associated with HIV-1 resistance encode a variety of immune molecules such as MHC class I, chemokine receptor (CCR) 5, KIR, and TLRs. For example, the HLA-B*27, B*57, the Bw4 alleles as well as heterozygosity at these class I loci are associated with slower disease progression [116]. However, the effect of these alleles on HIV-1 disease progression is limited to Caucasian and African populations [117]. In the Japanese population, HLA-B*52, B*67, and C*12 have been associated with a lower viral load [117]. East Asian populations also have a high proportion of individuals carrying a deletion in the A3B coding region, which increases susceptibility to HIV-1 infection [118]. There are also significant population differences in the CCR5 Δ32 mutation that is associated with resistance to HIV-1 [119]. The Δ32 mutation, for example, is only found in European, West Asian, and North African populations and shows a north-to-south decline [120]. Individuals homozygous for this mutation have a non-functional CCR5 receptor, and protection has been observed in both Δ32 homozygous and heterozygous individuals [120].

Similarly, breed differences in resistance to SRLV, as well as polymorphisms in ovine TLR 7, 8, CCR5 and MHC genes, have been found to be associated with resistance to SRLV [114,121,122,123,124]. For example, a higher proportion of polymorphisms in the ovine TLR 7 and 8 leucine rich repeat have been identified in MVV-infected sheep [121], and a deletion in the ovine CCR5 gene was associated with a reduced proviral load in rambouillet, Polypay and Columbia breeds [124]. Given these genetic parameters, it may be possible to selectively breed sheep and goats to have enhanced SRLV resistance. Many of the genetic polymorphisms associated with HIV-1 resistance were identified using genome-wide association studies (GWAS). To date, few sheep GWASs, and no goat GWASs have been carried out to identify genes associated with SRLV resistance. One possible reason for this may be due to difficulty in reliably identifying the phenotype, using current diagnostic methods. For example, the agar gel immunodiffusion (AGID) test and the enzyme-linked immunosorbent assay (ELISA) are widely used to detect SRLV infection [125]. Both of these tests, however, depend on the presence of host antibodies that are specific to SRLV, which are influenced by variables such as age and health status of the animal [126]. It is also possible that the AGID and ELISA tests may be influenced by genetic differences among SRLV strains [7]. Polymerase chain reaction (PCR) is also being used to test for the presence of viral RNA and proviral DNA in different tissues. This test is more sensitive than serological testing, however, problems with primer binding heterogeneity can make detection difficult [127] and the increased cost of PCR makes this relatively inaccessible to most producers [11]. Despite these challenges, a recent ovine GWAS identified a transmembrane protein gene, TMEM154, as a candidate gene for SRLV resistance [128]. The role of this protein is currently unknown, but it is expressed at high levels in B cells and monocytes, which suggests that it may be of immunological importance [128]. Breed differences have also been identified for the TMEM154 gene, the Dalsebred, Herdwick, and Rough Fells breeds had a higher allele frequency for the TMEM154 mutation that is associated with SRLV resistance [123]. An additional ovine GWAS identified another transmembrane protein, TMEM38A, as a possible gene associated with SRLV resistance, whereas the DPPA2 gene was associated with susceptibility [129]. The DPPA2 gene is involved with embryonic lung development and suggests that altered lung development in sheep may increase susceptibility to SRLV infections [129].

Although breeding livestock for enhanced disease resistance is becoming an increasingly popular means of improving animal health, in the context of SRLV infections, using genomic selection to breed for SRLV resistance may not be practical. As observed in HIV-1 studies, resistance or slower disease progression is a polygenic trait, involving a complex interaction between a variety of different innate and acquired immune genes [130,131]. It may, however, be possible to breed for resistance using phenotypic rather than genotypic selection. In sheep, helminth resistance is also a polygenic trait and resistant sheep have been bred based on phenotypic parameters such as fecal egg count [132]. However, it is unclear if breeding for resistance to one disease will increase susceptibility to others. Also, if SRLV resistance was introduced based on phenotypic selection, it is unclear how the virus will adapt. Since SRLV mutation rates are high and the virus already mutates in response to immune-based selection pressure, it is possible that the virus will quickly adapt, rendering the breeding program redundant.

An additional approach to breeding for SRLV resistance is by breeding for overall enhanced immune responses (EIR). This approach involves measuring CMIR and AbMIR in response to various antigens. A recent GWAS in dairy cattle found several single-nucleotide polymorphisms (SNPs) in the MHC locus that were associated with both AbMIR and CMIR [133]. In goats, the MHC haplotype Be10-D2 was associated with rapid seroconversion and higher antibody titers compared to the Bel-D5 haplotype [114]. Given this association, it is possible that further investigation and SNP discovery will allow for the identification of EIR sheep and goats that readily control SRLV disease progression.

## 5. Conclusions

The immune response to lentiviral infections is a complex and dynamic response, and to date very little is known about how the immune system responds to these infections. Extensive research on HIV-1 has greatly contributed to our knowledge of the dynamics of the host–virus interaction; however, HIV and SRLV, despite their similarities are two distinct viruses and extrapolating knowledge from HIV research must be approached with caution. It is evident from the research presented here that gaps exist in our knowledge of SRLV, and before exploring the possibility of breeding for resistance, extensive research needs to be done to better understand SRLV immune responses. These knowledge gaps primarily lie in the areas that focus on how SRLV modulate the host immune response and the dynamics of early infection. As previously discussed, for example, the Th1/Th2 paradigm appears to apply to SRLV infection; however, if in fact SRLV infection steers macrophage polarization towards a M2 phenotype favoring the Th2 immune response, then one would expect that the disease would progress rapidly and clinical disease would be apparent in a large proportion of animals. However, it often takes years before clinical SRLV infection becomes apparent. Therefore, future studies should firstly investigate how SRLV infection alters macrophage function as a whole. To start, cytokine production and intracellular signaling should be investigated. It would also be beneficial to investigate how endogenous stress hormone levels affect disease resistance and progression, and how host macrophages respond to SRLV during co-infection with other pathogens. Since macrophages play an important role as both innate effector cells and antigen presenting cells, an improved understanding of SRLV-infected macrophage function will improve our understanding of how SRLV is controlled during early infection, how the acquired immune response is induced, and how SRLVs modulate the host immune response.
